# Genome Sequences of Two Microcystis aeruginosa (*Chroococcales*, *Cyanobacteria*) Strains from Florida (United States) with Disparate Toxigenic Potentials

**DOI:** 10.1128/MRA.00844-20

**Published:** 2020-09-24

**Authors:** Forrest W. Lefler, Maximiliano Barbosa, David E. Berthold, H. Dail Laughinghouse

**Affiliations:** a Agronomy Department, University of Florida, Institute of Food and Agricultural Sciences, Fort Lauderdale Research and Education Center, Davie, Florida, USA; Georgia Institute of Technology

## Abstract

Here, we report the draft genomes of two Microcystis aeruginosa strains, i.e., M. aeruginosa BLCC-F108, which was isolated from a toxic bloom in eutrophic waters in Lake Okeechobee (Florida, USA), and M. aeruginosa BLCC-F158, which was isolated from mesotrophic waters in Lake Tohopekaliga (Florida, USA). Genomic analyses show disparate toxin potentials for these two strains.

## ANNOUNCEMENT

Microcystis aeruginosa (Kützing) Kützing is a cosmopolitan, freshwater, bloom-forming cyanobacterium that is notorious for its role in cyanobacterial harmful algal blooms (cyanoHABs). The occurrence of cyanoHABs can cause discoloration of the water and surface scums and is driven primarily by increases in nutrients (e.g., nitrogen and phosphorus) from point and nonpoint sources and internal cycling (1). Microcystis aeruginosa is also capable of producing toxic bioactive compounds that can be concentrated in water, sediments, animals, and plants, representing both environmental and public health threats (1). Because cyanoHABs are increasing in frequency, intensity, and duration globally (2), it is essential to understand their genomic diversity.

Microcystis aeruginosa BLCC-F108 was isolated from a subsurface grab sample from a toxic bloom in Lake Okeechobee, Florida, in April 2019, and M. aeruginosa BLCC-F158 was isolated from a subsurface grab sample from Lake Tohopekaliga, Florida, in January 2020. For isolations, samples were spread onto BG11 agar plates (3) and grown under a 12:12 light/dark cycle. Individual colonies were picked, grown in liquid BG11 medium, and visually checked by light microscopy for contamination to ensure unicyanobacterial cultures. Because it is difficult to achieve axenic cyanobacterial cultures and heterotrophic bacteria are well-known inhabitants of the *Microcystis* mucilaginous sheath (4), only unicyanobacterial cultures were achieved. An enzyme-linked immunosorbent assay (ELISA) kit (Eurofins Abraxis, Warminster, PA, USA) was used, following the manufacturer’s protocols, to evaluate microcystin production for the two strains. M. aeruginosa BLCC-F108 was confirmed to be a microcystin producer, while M. aeruginosa BLCC-F158 did not produce microcystins.

DNA was extracted using a DNeasy plant minikit (Qiagen, Germantown, MD, USA). For genome sequencing, libraries were prepared using the Illumina TruSeq library construction kit, and 2 × 150-nucleotide paired-end reads were generated with an Illumina HiSeq instrument. Default parameters were used for all software unless otherwise noted. Trimming was performed using fastp v0.20.1 (5), and quality was checked using FastQC v0.11.9 (6). Raw reads were *de novo* assembled using SPAdes v3.14.1 (7) with meta parameters. Contigs were then binned using MaxBin2 v2.2.4 (8), the bin corresponding to cyanobacteria was extracted, and the completeness and contamination were assessed using CheckM v1.0.18 (9) before annotation with the Prokaryotic Genome Annotation Pipeline (PGAP) v4.12 (10). The presence of secondary metabolite biosynthetic gene clusters (BCGs) was assessed using antiSMASH v5.1.2 (11). The draft genome size of M. aeruginosa BLCC-F108 is 5,037,850 bp, and the draft genome size of M. aeruginosa BLCC-F158 is 5,168,077 bp. Complete results can be found in Table 1.

**TABLE 1:** Genome data of two Microcystis aeruginosa strains

Feature	Data for:	
	Microcystis aeruginosa BLCC-F108	Microcystis aeruginosa BLCC-F158
Assembly size (bp)	5,037,850	5,168,077
No. of reads	9,909,010	8,564,402
No. of contigs	487	512
*N*_50_ (bp)	17,855	27,581
G+C content (%)	42.60	42.80
No. of coding sequences	4,428	4,699
Completeness (%)	99.23	99.89
Contamination (%)	0.51	0.62
No. of tRNAs	41	42
Coverage (×)	125	235
SRA accession no.	SRR12598970	SRR12599144
GenBank accession no.	JACEGB000000000	JACEGC000000000

Several BCGs were identified on the basis of identity to known BCGs within the antiSMASH database. M. aeruginosa BLCC-F108 was found to have a 100% match to a microcystin gene cluster, a 91% match to a piricyclamide gene cluster, a 100% match to a micropeptin K139 gene cluster, and a 78% match to an aeruginosin 98-A gene cluster. M. aeruginosa BLCC-F158 was found to have a 100% match to toxic compound anabaenopeptin, micropeptin K139, and microviridin J gene clusters, as well as an 80% match to a microviridin B gene cluster.

### Data availability.

The whole-genome shotgun projects for Microcystis aeruginosa BLCC-F108 and M. aeruginosa BLCC-F158 have been deposited in DDBJ/ENA/GenBank under the accession numbers JACEGB000000000 and JACEGC000000000, respectively. The versions described in this paper are the first versions, JACEGB010000000 and JACEGC010000000, respectively. The GenBank BioProject, BioSample, and SRA accession numbers for M. aeruginosa BLCC-F108 are PRJNA647122, SAMN15575897, and SRR12598970, respectively, and those for M. aeruginosa BLCC-F158 are PRJNA647120, SAMN15576007, and SRR12599144, respectively.
